# A genomic view of environmental and life history controls on microbial nitrogen acquisition strategies

**DOI:** 10.1111/1758-2229.13220

**Published:** 2023-12-06

**Authors:** Linta Reji, Romain Darnajoux, Xinning Zhang

**Affiliations:** ^1^ Department of Geosciences Princeton University Princeton New Jersey USA; ^2^ High Meadows Environmental Institute Princeton University Princeton New Jersey USA

## Abstract

Microorganisms have evolved diverse strategies to acquire the vital element nitrogen (N) from the environment. Ecological and physiological controls on the distribution of these strategies among microbes remain unclear. In this study, we examine the distribution of 10 major N acquisition strategies in taxonomically and metabolically diverse microbial genomes, including those from the Genomic Catalogue of Earth's Microbiomes dataset. We utilize a marker gene‐based approach to assess relationships between N acquisition strategy prevalence and microbial life history strategies. Our results underscore energetic costs of assimilation as a broad control on strategy distribution. The most prevalent strategies are the uptake of ammonium and simple amino acids, which have relatively low energetic costs, while energy‐intensive biological nitrogen fixation is the least common. Deviations from the energy‐based framework include the higher‐than‐expected prevalence of the assimilatory pathway for chitin, a large organic polymer. Energy availability is also important, with aerobic chemoorganotrophs  and oxygenic phototrophs notably possessing ~2‐fold higher numbers of total strategies compared to anaerobic microbes. Environmental controls are evidenced by the enrichment of inorganic N assimilation strategies among free‐living taxa compared to host‐associated taxa. Physiological constraints such as pathway incompatibility add complexity to N acquisition strategy distributions. Finally, we discuss the necessity for microbially‐relevant spatiotemporal environmental metadata for improving mechanistic and prediction‐oriented analyses of genomic data.

## Graphical abstract

Microorganisms employ a variety of strategies to obtain the critical element nitrogen from the environment. This work utilizes a genomic approach to examine environmental and metabolic controls on microbial nitrogen acquisition strategies and discusses a framework to examine the relative preference for various nitrogenous compounds in diverse environments.

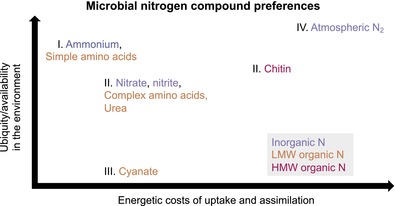

## INTRODUCTION

Nitrogen (N) is a critical element for life, and microorganisms have evolved diverse strategies for acquiring N from the environment. These N acquisition strategies target various N forms, including the inorganic forms dinitrogen (N_2_) gas, ammonia/ammonium, nitrite and nitrate; nitrogenous organic compounds such as urea, cyanate and amino acids; as well as large N‐containing polymers such as chitin and peptidoglycan. The energetic costs of uptake and assimilation (i.e., ATP, reducing equivalents) vary across compound classes, and this is thought to regulate N substrate preferences among microbes (Norman & Friesen, 2017). Ample and diverse lines of evidence indicate that microbes downregulate energetically expensive N assimilation pathways such as biological nitrogen fixation (BNF) or chitin depolymerization when small, more cheaply assimilated fixed N compounds are available (e.g., Allison & Macfarlane, 1992; Bidochka & Khachatourians, 1993; Geisseler et al., 2010; Mulholland et al., 1999). Whether microbial genomic architectures reflect these differences in substrate preferences remains unclear.

Cellular costs of uptake and assimilation vary depending on the molecular size and redox state of the N compound. N assimilation in most microbes proceeds via the incorporation of ammonium into carbon skeletons through the glutamine synthase–glutamate synthase (GS–GOGAT) cycle, producing glutamine and glutamate, which are incorporated into cellular anabolism. The uncharged species ammonia (NH_3_) can enter the cell via diffusion when it is abundant in the environment (e.g., alkaline conditions; Kleiner, 1981). Specialized transporters are widely used by microbes to transport both NH_3_ and the protonated form ammonium (NH_4_^+^) into the cell (Javelle et al., 2006). The GS–GOGAT pathway consumes 1 ATP per molecule of NH_4_^+^.

Amino acids are another major class of low molecular weight (LMW) organic N compounds available for microbes in the environment. While amino acids can be assimilated relatively easily to cellular macromolecules, their uptake can involve energetic investment in the form of proton/sodium ion motive force (PMF/SMF) and/or ATP (Saier Jr., 2000). Most amino acid transporters are PMF‐ or SMF‐dependent secondary active transporters (Saier Jr., 2000) (i.e., move substrates against the concentration gradient while transporting other solutes down their concentration gradient). As amino acids can also serve as a source of carbon and sulphur, and as compatible solutes for adapting to osmotic stress, microbial uptake of amino acids does not necessarily reflect only their nitrogen strategy. In terms of energetic investment, amino acid assimilation should be equally, if not more favourable, compared to ammonium given the minimal post‐uptake modifications required. Experimental evidence indicates that dissolved amino acids can repress ammonium uptake and assimilation, at least in heterotrophic microbes (Jørgensen et al., 1999; Kirchman et al., 1989).

Assimilating N sources other than NH_3_/NH_4_^+^ or amino acids incurs additional energetic investments in terms of specialized transporters for uptake as well as intracellular reduction of the substrate to NH_4_^+^. For example, assimilating one molecule each of nitrite (NO_2_^−^) and nitrate (NO_3_^−^) consumes six and eight reducing equivalents, respectively. As a result, even in marine waters with plenty of NO_3_^−^, many phytoplankton preferably use less abundant forms of N such as ammonium, amino acids and urea (reviewed in Díez et al., 2023; Hutchins & Capone, 2022). BNF, the process by which microbes assimilate atmospheric N_2_, requires 8 reducing equivalents and 16 ATPs per mole of N_2_ fixed. Additional indirect costs for assimilation include making and maintaining the complex enzymatic machinery required for each process, particularly BNF. Acquiring high molecular weight (HMW) organic N sources also require significant metabolic energy input due to the diverse and bulky nature of these compounds. Microbes must synthesize not only specific transporters targeting these compounds, but also diverse extracellular enzymes to break down large polymers in the extracellular environment prior to uptake.

Based on the relative difficulty with which microbes can access diverse N sources, as outlined above, Norman and Friesen (2017) proposed a N acquisition strategy for free‐living N_2_‐fixing microbes (diazotrophs) under fixed N limitation, which postulates a preference for LMW organic N over BNF, followed by HMW organic N. Accounting for the relative energy investments required for accessing, transporting and assimilating different N sources, this ‘LAH strategy’ (which stands for ‘LMW N–atmospheric N_2_–HMW N’) proposes the following order of N compound preferences for free‐living diazotrophs: ammonia/ammonium > nitrite/nitrate > LMW organic N > BNF > HMW organic N. Norman and Friesen (2017) indeed found evidence from pure culture growth experiments and genomes that several lineages of free‐living soil diazotrophs can access the HMW‐N pool (primarily via extracellular protein degradation). Under the LAH framework and the differential energetic investments involved in the assimilation of various N sources, it may be presumed that the relative distribution of N acquisition strategies among microorganisms follow their respective substrate preferences.

The broader validity of the LAH strategy remains to be tested. Notably, the realized cost of N acquisition likely varies depending on a microbe's environmental context and life history adaptations (i.e., substrate flux, mode of metabolism, resource allocation, motility, etc.). For instance, the dynamic nature of the bioavailable N pool as well as spatial heterogeneity of geochemistry in structured environments such as soils (Smith et al., 2022) may shape the relative distributions of N acquisition strategies within natural microbial communities. Several outstanding questions in this regard are: (i) How prevalent are different N acquisition strategies among microbes across environmental systems? (ii) Are specific pathway combinations preferred over others? (iii) How do life history traits (e.g., mode of metabolism, resource allocation, motility, etc.) and environmental context affect the number of N acquisition pathways present in an organism?

To address these questions, we conducted a large‐scale genomic study examining the distribution of 10 major N acquisition strategies across microbial genomes with taxonomic, metabolic and habitat diversity. While the distribution and prevalence of a single pathway has been examined previously (e.g., nitrogen fixation genes among fully sequenced genomes in Dos Santos et al., 2012), a comprehensive analysis of various N acquisition strategies across the diversity of microbial life is so far lacking. Here, we utilize >10,000 metagenome‐assembled genomes (MAGs) from the Genomic Catalog of Earth's microbiomes (GEMs) dataset, representing diverse environmental systems (Nayfach et al., 2021). We also conducted a targeted analysis of six microbial clades chosen based on diverging metabolic and evolutionary relatedness, including complete genomes of the phylum *Cyanobacteria*, genus *Clostridium* and the orders *Rhizobiales*, *Desulfovibrionales*, *Methanobacteriales* and *Methanococcales*. The presence of both diazotrophic and non‐diazotrophic lineages within each clade enabled a more comprehensive examination of N acquisition capabilities. In each genome set, we employed a marker gene‐based approach to systematically catalogue the prevalence of the following 10 major N acquisition strategies: nitrogen fixation, ammonium uptake, ferredoxin‐dependent nitrite reduction; NADH‐dependent nitrite reduction; assimilatory nitrate reduction; urea uptake; cyanate uptake; chitin depolymerization; assimilation of chitin oligomers (i.e., beta‐*N*‐acetyl hexosamine); and amino acid uptake. We then examined the influence of metabolic adaptations and environmental context on the distribution of these N acquisition strategies amongst the diverse groups of microbes.

## EXPERIMENTAL PROCEDURES

### Compiling and pre‐processing genomes

Genome assemblies from the GEMs dataset were downloaded from https://portal.nersc.gov/GEM/ (Nayfach et al., 2021). The original set of 52,515 MAGs was filtered to only retain those meeting the MIMAG high quality genome criteria (Bowers et al., 2017). This resulted in 9143 MAGs. In addition to the GEMs set, complete genome sequences of organisms belonging to five selected clades—*Cyanobacteria*, *Clostridium*, *Rhizobiales*, *Desulfovibrionales*, *Methanobacteriales* and *Methanococcales*—were downloaded from the NCBI RefSeq database using genome_updater (https://github.com/pirovc/genome_updater) in March 2022. All genome assemblies were analysed using Prodigal (Hyatt et al., 2010) to predict protein‐coding sequences.

### Examining N acquisition strategy prevalence across genomes

We examined the incidence of 10 N acquisition strategies using specific functional genes indicative of each strategy. Marker gene‐based surveys can yield false‐positive results, particulary if genes encoding critical enzyme subunits or structural components are absent from the genome. To mitigate such artefacts, we focused on functionally critical elements of the key enzyme associated with each pathway. The following pathway‐marker gene combinations were used in our analysis (Table S1): ammonium uptake (marker gene: ammonium permease, AmtB); BNF (nitrogenase, NifHDK); Ferredoxin (Fd)‐dependent nitrite assimilation (Fd‐nitrite reductase, NirA); NADH‐dependent nitrite assimilation (NADH‐nitrite reductase, NirBD); nitrate assimilation (assimilatory nitrate reductase, NasA co‐occurring with an assimilatory nitrite reductase); cyanate assimilation (cyanase, CynS); urea assimilation (urease, UreC), chitin depolymerization (chitinase), *N*‐acetyl hexosamine assimilation (*N*‐acetyl hexosaminidase) and amino acid uptake (various amino acid transporters).

For strategies other than amino acid uptake, reference protein sequences for each pathway marker were obtained from the NCBI RefSeq protein database (Table S1). Reference sequences belonging to five distinct families of amino acids (all within the APC superfamily) were obtained from the Transporter Classification Database (TCDB; Saier et al., 2021; Saier Jr et al., 2006; Table S1).

For each marker protein, multiple sequence alignments were generated using MAFFT (Katoh et al., 2002). These alignments were then used to generate profile HMMs for each protein via hmmbuild in HMMER (http://hmmer.org/). Following this, hmmsearch was used to identify homologues of each marker protein across all genomes. The E‐value thresholds for hit identification were adjusted based on BLASTp search results against the UNIPROT/SWISSPORT databases (UniProt Consortium, 2023).

The putative hits for each marker protein were then aligned with reference sequences using MAFFT (Katoh et al., 2002), and individual phylogenetic trees were generated using FastTree (Price et al., 2009, 2010). The trees were inspected in Taxonium (Sanderson, 2022) and manually curated. Curation involved BLASTp searches of sequences from each major phylogenetic cluster against the RefSeq as well as the Uniprot/Swissport databases. Putative amino acid transporter hits were searched against the TCDB (Saier et al., 2021; Saier Jr et al., 2006) to assess homology. False positives identified based on homology searches were removed from the final list of hits for each marker. Curation was particularly challenging for nitrate and nitrite reductases as the hits often included diverse kinds of oxidoreductases. To be conservative, we discarded all sequences that could not be confidently classified as a NasA or NirA. Curated results are summarized in Table S2.

### Statistical analyses

All statistical analyses were carried out in R (v4.1.2). Of the 9143 genomes in the filtered GEMs dataset, one did not have a Domain‐level taxonomic identification. Additionally, 274 genomes did not contain homologues of any of the 10 examined pathways. Those missing any of the 10 examined pathways were not analysed further as it was impossible to determine if this was an artefact of genome incompleteness. The final filtered GEMs set included 8868 genomes, consisting of 8696 bacteria and 172 archaea.

For comparisons involving metabolic adaptations, we classified the genomes as ‘aerobic’, ‘facultatively anaerobic’, ‘anaerobic’ and ‘phototrophic’. The genomes were also classified as ‘photoautotrophic’, ‘photoheterotrophic’, ‘chemoautotrophic’, ‘chemoorganotrophic’ and ‘mixotrophic’. These codings were assigned based on expected metabolic strategy within a conserved taxonomic level based on what has been reported in the literature (e.g., all genomes within class *Cyanobacteria* were coded as oxygenic photoautotrophs). Lineages lacking physiologically characterized members were excluded from the GEMs dataset, which resulted in a final set of 4826 genomes for metabolism‐based analyses (Table S3).

Differences in N strategy incidence/prevalence distributions across various metadata variable groupings were statistically tested using Kruskal–Wallis test followed by post‐hoc pairwise comparisons using the Wilcoxon Rank Sum test. Generalized mixed effects linear models were used to compare associations between various N acquisition strategy metrics and metadata variables, controlling for taxonomic relatedness between genomes. Dimensionality reduction was carried out by using prcomp (https://www.rdocumentation.org/packages/stats/versions/3.6.2/topics/prcomp) and logisticPCA (Landgraf & Lee, 2015).

## RESULTS AND DISCUSSION

### Genomic patterns of microbial N acquisition indicate importance of ammonium and organic N compounds

Based upon the expected energetic costs of uptake and assimilation, as proposed under the LAH framework, microbial N acquisition is expected to follow a predictable order of compound preferences (Norman & Friesen, 2017). More easily assimilated compounds such as inorganic N (ammonium, nitrite and nitrate) and LMW organic N compounds (amino acids, urea and cyanate) are preferred over BNF and HMW organic N compounds. Subsequently, it can be inferred that the incidence of the various N acquisition strategies in microbial genomes tracks the relative order of N compound preferences. For example, inorganic and LMW organic N uptake pathways should be more common compared to BNF and HMW‐N uptake pathways across microbial genomes. To test this prediction, we utilized the GEMs dataset, which includes 52,515 genomes assembled from 10,450 metagenomes originating from diverse environments (Nayfach et al., 2021).

**FIGURE 1:**
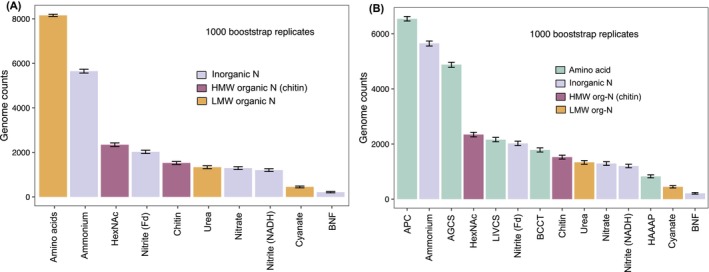
Amino acid and ammonium acquisition are the predominant N acquisition strategies in microbial genomes; and high molecular weight (HMW) organic N assimilation may be more common than inorganic N acquisition. (A) Prevalence of the various N‐compound acquisition strategies among genomes in the filtered GEMs dataset. BNF, biological nitrogen fixation; HexNAc, *N*‐acetyl‐hexosamine, a compound class that includes chitin monomers. A non‐parametric bootstrapping approach (1000 bootstrap replicates) was used to reduce biases from uneven sampling depths. Bars indicate the bootstrapped median number of genomes containing each strategy; error bars indicate the 95% bootstrap confidence intervals around the median. (B) Prevalence patterns presented in panel (A), with greater resolution into the relative prevalence of amino acid transporter families. AGCS, alanine/glycine:cation symporter; APC, Amino Acid‐Polyamine‐Organocation family; BCCT, betaine/carnitine/choline transporter; HAAAP, hydroxy/aromatic amino acid permease; LIVCS, branched chain amino acid:cation symporter. The APC subfamily consists of amino acid transporters not included within the remaining four families.

In examining the quality‐filtered GEMs genomes, amino acid uptake emerges as the most prevalent among the 10 N strategies examined, closely followed by ammonium uptake (Figure 1A). This likely reflects the fact that ammonium and amino acid incorporation necessitates minimal downstream modification of the acquired substrate. Some forms of amino acids may be preferred over others, however. Our analysis included five subfamilies of amino acid transporters as defined within the TCDB (Saier et al., 2021; Saier Jr et al., 2006): betaine/carnitine/choline (BCCT) family, alanine/glycine:cation symporter (AGCS) family, branched chain amino acid:cation symporter (LIVCS) family; hydroxy/aromatic amino acid permease (HAAAP) family; and the APC family consisting of those not falling within any of the remaining four families. Among these, BCCT, LIVCS and HAAAP families target more complex amino acid forms compared to AGCS. Indeed, AGCS transporters were significantly more prevalent than branched‐chain/aromatic amino acid transporters (Figure 1B). Thus, these results confirm that the extent of downstream modification of the substrate before incorporation into anabolism is a key determinant of N‐substrate preference.

Another notable result from the GEMs analysis is the higher‐than expected prevalence of genes involved in chitin depolymerization and the uptake of chitin monomers (i.e., *N*‐acetyl‐hexosamine, HexNAc). Chitinase and *N*‐acetyl‐hexosaminidase genes are more common compared to those for nitrite, nitrate, urea and cyanate assimilation (Figure 1). These patterns were robust to multiple resampling approaches performed to reduce taxonomic sampling bias (Figure S1a–d). However, we do not fully discard biases resulting from overrepresentation of certain habitat types (e.g., human and other host microbiomes) as well as underrepresentation of poorly assembling lineages such as SAR11 in the GEMs dataset.

Chitin‐related genes were more prevalent than genes for assimilation of smaller organic N compounds such as urea and cyanate (Figure 1; Figure S1). The prevalence of chitinases might reflect its widespread availability as the second most abundant biopolymer in nature (Jiménez‐Gómez & Cecilia, 2020). Yet it remains unclear why chitin degradation may be more prevalent than urea assimilation. This could be related to the relative stability of each compound in the environment (i.e., urea readily dissociates into ammonia and carbon dioxide). For cyanate, however, the relatively low environmental availability of this compound (up to ~10,000‐fold lower availability compared to ammonium across habitat types [Mooshammer et al., 2021]) may be a contributing factor.

Among the inorganic‐N strategies, excluding ammonium uptake, ferredoxin‐dependent nitrite reduction appears to be the most prevalent strategy (Figure 1; Figure S1). As would be predicted based upon the energetic cost of assimilation paradigm, BNF is the least prevalent strategy among the GEMs genomes. This result notably contrasts with predictions of the LAH framework, which has suggested that atmospheric N_2_ assimilation might be preferred over HMW‐N acquisition.

**FIGURE 2:**
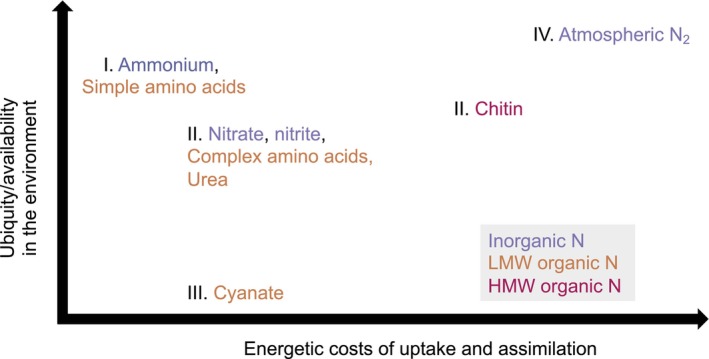
Constraints on microbial N‐compound preferences indicated by the genomic analysis. The order of compound preferences likely follows the energetic costs of assimilation as well as relative compound availabilities, as discussed in the text. Roman numerals indicate inferred order of compound preferences based on the GEMs analysis. Additional controls on the incidence of N strategies not depicted here includes physiological factors such as metabolic toxicity (i.e., oxygen toxicity of BNF) and pathway incompatibility (e.g., nitrite interfering with sulphite reduction in sulphate reducers). BNF, biological nitrogen fixation; GEM, Genomic Catalog of Earth's microbiomes; N, nitrogen.

Hence, the genomic patterns observed in the GEMs dataset (Figure 1) indicate a slightly different order of N‐compound preferences compared to the predictions of the LAH framework (Figure 2). Simple amino acids may be equally if not more preferred over ammonium given similar energetic investments in the uptake/assimilation of these compounds. HMW organic‐N assimilation may be more prevalent than recognized, compared to specialized strategies targeting LMW organic compounds such as urea and cyanate. As these patterns indicate, energetics of uptake/assimilation do not sufficiently explain the relative distributions of the different strategies, suggesting more complex environmental and life history controls on pathway distributions. We examine these next.

### The abundance of microbial N acquisition strategies scale with genome size and metabolic energy yield

Given the differential direct and indirect energetic and cellular costs of N acquisition strategies, we hypothesized that the distribution and diversity of the different strategies would vary as a function of metabolic energy yield. Metabolic modes yielding higher free energy per reaction (e.g., oxygen respiration) likely result in higher cellular N demand (e.g., due to faster growth rate and associated faster turnover of cellular components) while at the same time affording the microbe the ability to maintain and use multiple uptake/assimilation pathways. In testing this hypothesis, we considered two fundamental aspects of the metabolic strategy of an organism: mode of respiration (or the lack thereof; classified as anaerobic, aerobic, phototrophic or facultative anerobic in our analyses) and energy metabolism (chemoorganotroph, chemoautotroph, photoautotroph, photoorganotroph and mixotroph). First, we assessed if larger genomes correspond with higher number of total N acquisition strategies. We then examined if the number of N acquisition strategies vary across different modes of metabolism.

**FIGURE 3:**
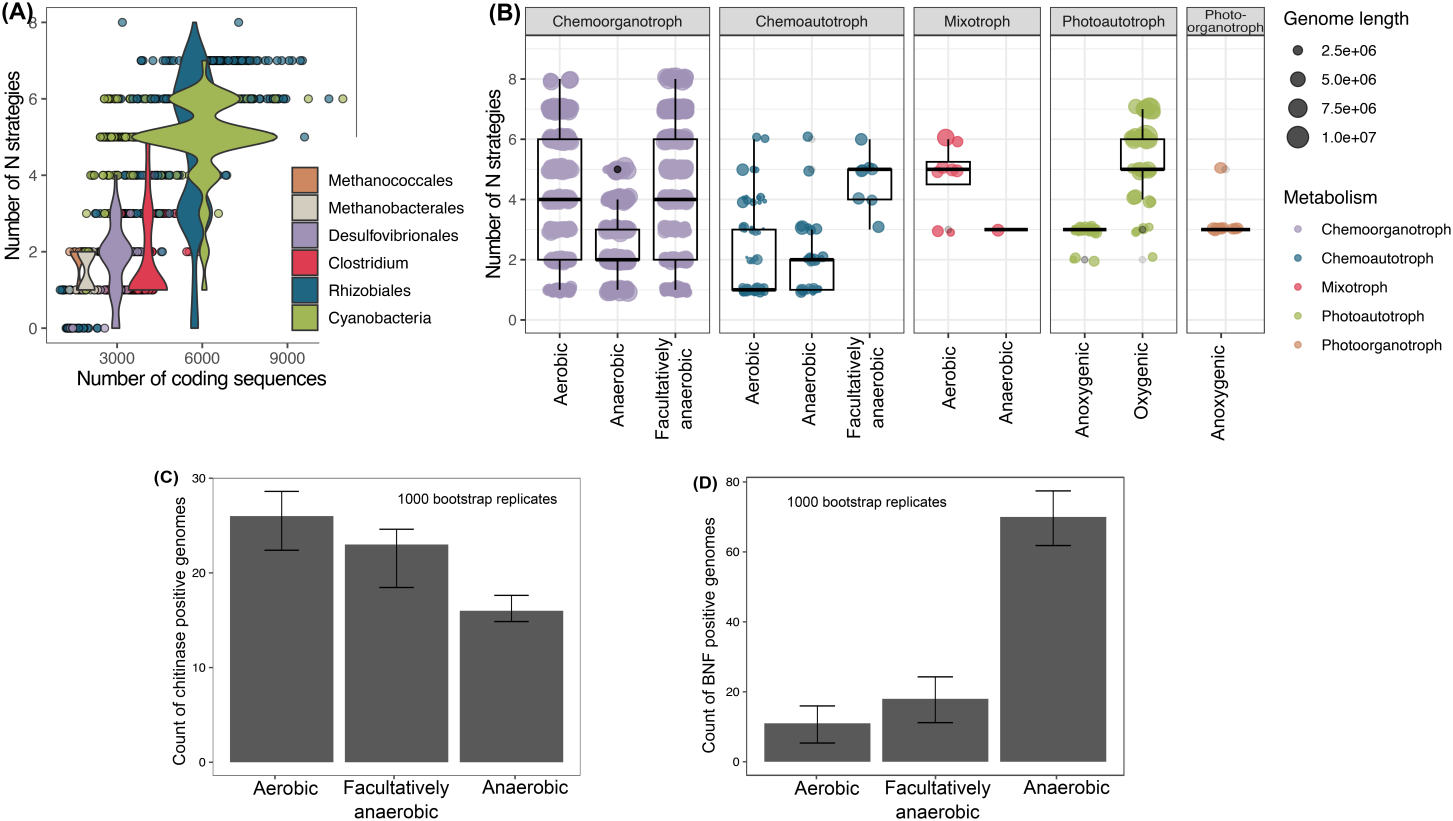
Diversity of N acquisition strategies scales with microbial genome size and correlates with aerobic respiration. (A) The number of N acquisition pathways detected versus the number of coding sequences in selected phylogenetic clades. The violin plots indicate the distribution of N‐pathway counts across clades. (B) Total number of N acquisition pathways across all genomes in the filtered GEMs set (*n* = 4749) partitioned by metabolic mode. (C,D) The number of (C) chitinase‐ and (D) BNF‐positive genomes across aerobic, anaerobic and facultatively anaerobic lineages in the filtered GEMs dataset. Error bars indicate 95% bootstrap confidence intervals around the median. BNF, biological nitrogen fixation; GEM, Genomic Catalog of Earth's microbiomes; N, nitrogen.

In analysing the N strategy distributions within a set of six microbial clades (selected based on the presence of diazotrophic and non‐diazotrophic members within each clade, and the diversity of central metabolic strategies)—*Cyanobacteria*, *Clostridia*, *Desulfovibrionales*, *Rhizobiales*, *Methanobacteriales*, *Methanococcales*—we observed that (i) the number of N assimilation pathways scaled with genome size, and (ii) lineages characterized by higher metabolic energy yield generally harboured a larger number of N acquisition strategies (Figure 3A). For example, aerobic or oxygen‐tolerant organisms (*Cyanobacteria* and *Rhizobiales*) generally possess higher number of N acquisition strategies than obligate anaerobes (*Clostridia*, *Desulfovibrionales* and methanogens; Figure 3A). This stands despite the typical habitat overlap between *Rhizobiales* and the non‐cyanobacterial clades, suggesting that any effect of physicochemical variability such as differential substrate diffusion and organic matter complexity may have a relatively weaker influence over N assimilation strategy distributions than the primary mode of metabolism. Additionally, the number of N assimilation strategies scale with the genome size among these clades (Figure 3A), suggesting that aerobic organisms with larger genomes generally have the potential to obtain N from a larger diversity of sources. Conversely, the ability to assimilate a larger diversity of N compounds also likely presents an opportunity to build larger genomes, establishing a reinforcing mechanism.

Expanding the analysis to the GEMs, we first examined genomes corresponding to the selected clades above within this larger set of MAGs. The GEMs set, after quality control (see Experimental procedures), did not include methanogenic archaeal genomes. Consistent with the patterns observed for the selected clades above, we observed a larger number of total N acquisition pathways in aerobic lineages with larger genomes (Figure S3). Observations for the overall filtered GEMs set (4749 genomes) align with these patterns as aerobic and facultatively aerobic organisms possess significantly more N acquisition strategies than anaerobic organisms (Figure 3B). Mode of metabolic energy generation appears to be an additional control on this as the diversity of acquisition strategies was significantly higher for (a) aerobic phototrophs over anaerobic phototrophs and (b) facultatively anaerobic chemoautotrophs over aerobic and obligately anaerobic chemoautotrophs (Figure 3B). In contrast, obligately anaerobic chemoorganotrophs have significantly lower diversity of N acquisition strategies compared to aerobic and facultatively anaerobic chemoorganotrophs (Figure 3B).

These results also indicate a significant correlation between genome length and the diversity of N acquisition strategies (linear regression on log [genome length] and the number of pathways; *p*‐value <2e‐16; Figure 3B; Figures S2 and S3), as seen in the case of the six selected clades (Figure 3A). In addition, there is a significant positive correlation between genome length and aerobic or facultatively anaerobic metabolisms (*p*‐values <2e‐16 and 1.82e‐05, respectively). In contrast, anaerobic respiration is significantly negatively correlated with genome length (*p*‐value <2e‐16). It then follows that oxygen‐respiring organisms, which tend to have larger genomes, harbour more diverse (larger number of) N acquisition strategies. While these results are expected at the broad scale of metabolic pathway distributions (e.g., larger genomes can accommodate higher number of metabolic pathways), this study confirms this principle for the acquisition of the key nutrient N.

Given the correlation between N pathway counts and metabolic energy yield (Figure 3), we can expect that higher cost N acquisition strategies will also correlate to metabolic energy yield. To test this hypothesis, we examined whether higher‐cost N acquisition pathways (i.e., BNF and chitin acquisition) are more prevalent among oxygen‐respiring organisms. Chitinase distribution was significantly positively associated with aerobic respiration (logistic regression, *p* < 0.001; Figure 3C). BNF, in contrast, was significantly negatively associated with aerobic respiration (glmm model; *p*‐value <2e‐16; Figure 3D). This result aligns with the notable oxygen toxicity of BNF, and further underscores that the prevalence of N acquisition strategies is not simply regulated by metabolic energy yield. Another striking example for metabolic compatability as a control is the typical absence of nitrite assimilation pathways among *Desulfovibrionales*, as nitrite interferes with their energy generation pathway due to its structural similarity with sulphite (Rajeev et al., 2015).

### Effect of habitat type on the prevalence of N acquisition strategies

Microbes living in association with a host (e.g., pathogens, parasites, symbionts and gut commensals) tend to undergo genome reduction due to various evolutionary forces pushing towards minimizing functional redundancy and bolstering the host–resident (inter)dependency (Andersson & Kurland, 1998; Noh, 2021). If the resident microbe is supplied with organic compounds by the host, independently acquiring N (particularly inorganic N) would be redundant and less efficient for the microbe. Therefore, we should expect to see genome reduction favouring the deletion of inorganic N acquisition pathways in host‐associated microbes. Similarly, free‐living taxa inhabiting nutrient‐rich environments can potentially afford to eliminate inorganic N acquisition strategies. We examined evidence for these expected patterns in the GEMs dataset.

**FIGURE 4:**
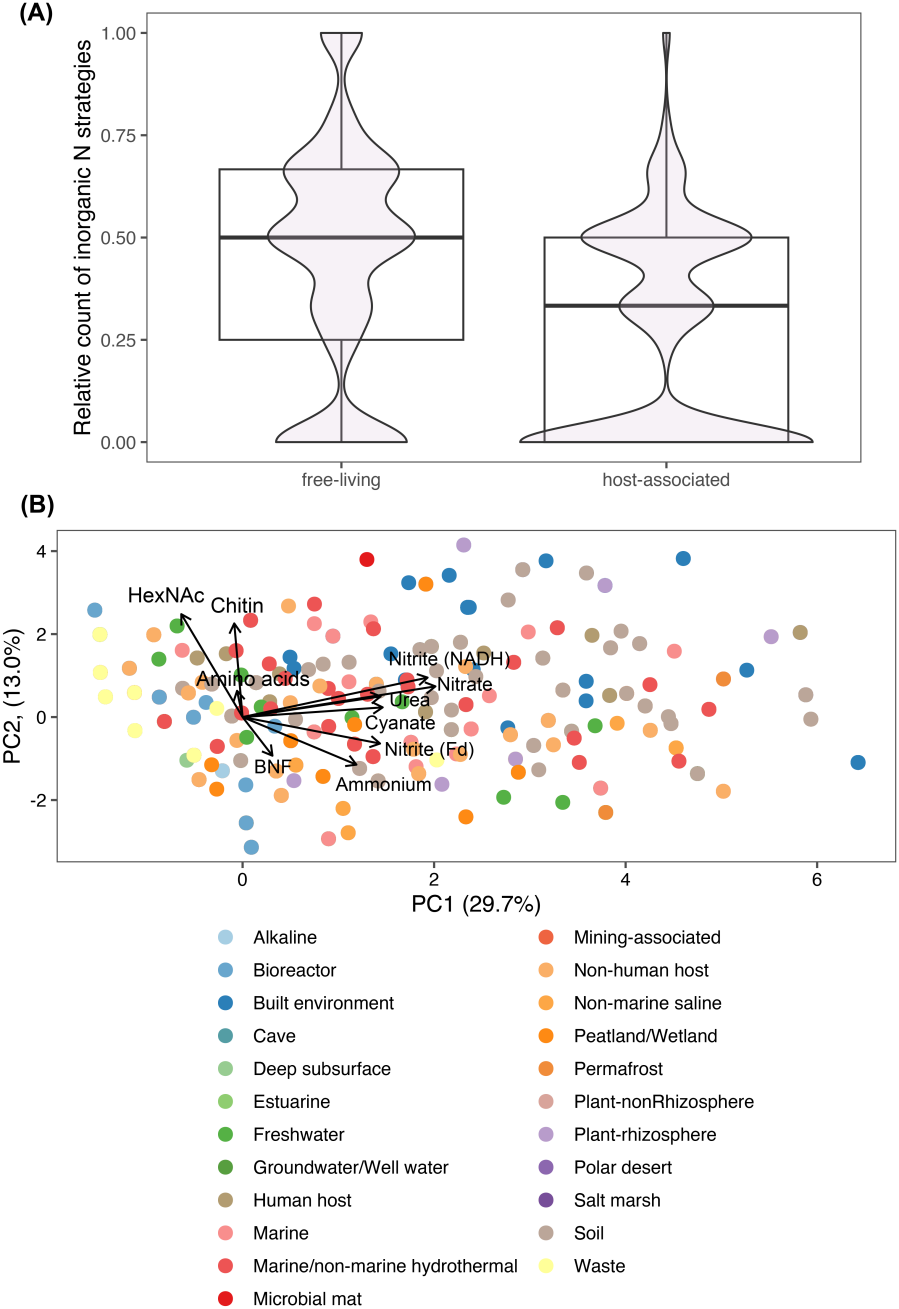
Relationships between environmental context and N acquisition strategy distributions. (A) Number of inorganic N acquisition strategies relative to the total number of N acquisition strategies in free‐living versus host‐associated taxa. (B) PCA analysis of the distribution of N acquisition strategies among the GEMs genomes across broad ecosystem categories. GEM, Genomic Catalog of Earth's microbiomes; N, nitrogen.

Indeed, taxa inhabiting host‐associated environments were found to be relatively more enriched in organic N acquisition pathways than free‐living taxa (Figure 4A; # *t* = 25.039, df = 3986.6, *p*‐value <2.2e‐16). This might reflect the greater availability of organic N compounds in the host environments, which makes inorganic N pathways redundant/futile. In addition, examining the relationship between inorganic N uptake and habitat type, we observed that inorganic N pathways are relatively less frequent among genomes obtained from high‐nutrient environments such as bioreactor or solid/liquid waste environments (Figure 4B; light blue and yellow points). Genomes assembled from built environments (predominantly city subway systems) frequently harbour chitin degradation potential, in addition to most LMW and inorganic N uptake strategies (Figure 4B; darker blue points). In addition to subway systems, chitin degradation is relatively more common among genomes assembled from permafrost and plant rhizosphere systems (Figure 4B; Figure S4), which may reflect greater relative availability of HMW N compounds in these systems.

We further tested associations between pathway incidence and ecosystem type using Poisson generalized mixed linear models with fixed effects for taxonomic relatedness at the family level. The results show significant associations between habitat type and N pathway distributions. There is a strong positive correlation between the number of organic N assimilation strategies and the following habitat types (corresponding *p*‐values in parentheses): plant rhizosphere (<2e‐16), built environment (i.e., city subways; <2e‐16), human host‐associated systems (1.9e‐10) and freshwater systems (7.46e‐14). Relatively weaker, yet significantly positive correlations were observed between the number of organic N strategies and aquatic marine systems (*p*‐value 0.0002), soil (0.004) and solid/liquid waste environments (0.001).

These observations most likely reflect adaptations of genome architecture to differential availability of fixed N compounds across environments. For example, symbiotic taxa in host‐associated environments may exhibit genome reduction, effectively lowering cellular energy requirements. This is observed in the case of the cyanobacterium *Candidatus Atelocyanobacterium* (UCYN‐A), which is able to fix N_2_ and supply fixed N to its host, but lacks additional N acquisition strategies, including ammonium uptake (Tripp et al., 2010). Similarly, many *Clostridia* genomes harbour chitinases and amino acid uptake systems yet lack additional pathways for N assimilation (including the typically prevalent ammonium uptake strategy; Table S2), and all of these turn out to be parasitic lineages that are likely adapted to N‐rich host environments.

Finally, we identify the lack of appropriate metadata for analyses as an outstanding challenge in deciphering habitat‐related patterns in pathway distributions. For example, environmental categories in the GEMs dataset are often too broad (e.g., ‘soil’, ‘freshwater’ or ‘marine’), which masks microhabitat diversity within each category. Environmental data at or close to the spatial scale of the microbe will be essential for making robust mechanism‐based inferences on N strategy distributions. Substrate availability and transport limitations can vary greatly across habitat types, which will add to the metabolic and ecological complexity of the microbial inhabitants.

## CONCLUSIONS

Our examination of the distribution of N acquisition strategies across microbial genomes reveal strong controls by the relative energetic costs of assimilation, which is influenced by specific metabolic adaptations to habitats and environmental conditions. The long standing energy cost framework explains the findings that assimilation of relatively cheap N compounds (ammonium and simple amino acids) dominates over all other strategies. Broader metabolic adaptations related to energy availability are also important: the higher free energy yield per mole of carbon from oxygen respiration likely affords the retention of larger, more versatile genomes in aerobic (and facultatively aerobic) organisms compared to anaerobic ones living at the bottom of the redox ladder (such as sulphate reducers and methanogens). Exceptions to this general pattern can result from additional controls including toxicity (i.e., pathway incompatibility) due to interactions between metabolites. For example, oxygen toxicity of BNF can explain the general prevalence of this strategy among obligate and/or facultative anaerobes. Similarly, the general absence of nitrite reduction in sulphate reducers may be explained by toxicity resulting from the structural resemblance between nitrite and sulphite. Additional habitat‐related aspects such as host‐associated versus free‐living, oligotrophic versus copiotrophic systems can exert additional, secondary controls on the incidence of N acquisition strategies. However, for robust testing of these hypotheses using genomic data, we need environmental data measured at microbially relevant scales.

Several additional N acquisition pathways that we did not analyse in this study include extracellular peptidases and carbohydrate‐active enzymes involved in the degradation of N‐containing polymeric compounds other than chitin. Furthermore, the presence of a gene/pathway complement in the genome does not necessarily indicate actual use of that pathway. For example, many organisms harbouring the genomic potential for BNF have not been shown to grow diazotrophically (e.g., *Holophaga foetida*, *Thermodesulfovibrio* sp.). This suggests additional complexity in factors controlling the realized niches of microbes that ultimately determines N compound preferences in different ecosystems. The selection pressure on genomes can be highly variable across ecological contexts, which can contribute to differential genome evolution patterns in different habitats. Our analyses shed light on some of the resulting patterns in N strategy distributions, and guide future experiments to examine the relative substrate preferences in organisms harbouring multiple N acquisition strategies. Finally, we emphasize the importance of accurate and microbially relevant metadata for enabling genomic analysis that support predictive modelling of microbial ecology and evolution.

## AUTHOR CONTRIBUTIONS

**Linta Reji:** Conceptualization (equal); data curation (lead); formal analysis (lead); investigation (lead); methodology (lead); validation (lead); visualization (lead); writing – original draft (lead). **Romain Darnajoux:** Conceptualization (equal); methodology (supporting); validation (supporting); writing – review and editing (equal). **Xinning Zhang:** Conceptualization (equal); funding acquisition (lead); project administration (lead); supervision (lead); writing – review and editing (equal).

## CONFLICT OF INTEREST STATEMENT

The authors declare no conflicts of interest.

## Supporting information


**DATA S1:** Supporting Information.

**DATA S2:** Supporting Information.

## Data Availability

All genomes analysed in this study are publicly available on the JGI genome portal or the NCBI RefSeq databases. R code used for statistical analyses and plotting has been uploaded to the GitHub repository: https://github.com/Linta‐Reji/ZhangLab_Reji2023_N_acquisition.

## References

[emi413220-bib-0001] Allison, C. & Macfarlane, G.T. (1992) Physiological and nutritional determinants of protease secretion by *Clostridium sporogenes*: characterization of six extracellular proteases. Applied Microbiology and Biotechnology, 37(2), 152–156.

[emi413220-bib-0002] Andersson, S.G. & Kurland, C.G. (1998) Reductive evolution of resident genomes. Trends in Microbiology, 6(7), 263–268. 9717214 10.1016/s0966-842x(98)01312-2

[emi413220-bib-0003] Bidochka, M.J. & Khachatourians, G.G. (1993) Regulation of extracellular N‐acetyl‐D‐glucosaminidase production in the entomopathogenic fungus *Beauveria bassiana* . Canadian Journal of Microbiology, 39(1), 6–12. 8439875 10.1139/m93-002

[emi413220-bib-0004] Bowers, R.M. , Kyrpides, N.C. , Stepanauskas, R. , Harmon‐Smith, M. , Doud, D. , Reddy, T.B.K. et al. (2017) Minimum information about a single amplified genome (MISAG) and a metagenome‐assembled genome (MIMAG) of bacteria and archaea. Nature Biotechnology, 35(8), 725–731. 10.1038/nbt.3893 PMC6436528 28787424

[emi413220-bib-0005] Díez, J. , López‐Lozano, A. , Domínguez‐Martín, M.A. , Gómez‐Baena, G. , Muñoz‐Marín, M.C. , Melero‐Rubio, Y. et al. (2023) Regulatory and metabolic adaptations in the nitrogen assimilation of marine picocyanobacteria. FEMS Microbiology Reviews, 47(1), fuac043. 10.1093/femsre/fuac043 36323406

[emi413220-bib-0006] Dos Santos, P.C. , Fang, Z. , Mason, S.W. , Setubal, J.C. & Dixon, R. (2012) Distribution of nitrogen fixation and nitrogenase‐like sequences amongst microbial genomes. BMC Genomics, 13, 162. 22554235 10.1186/1471-2164-13-162 PMC3464626

[emi413220-bib-0007] Geisseler, D. , Horwath, W.R. , Joergensen, R.G. & Ludwig, B. (2010) Pathways of nitrogen utilization by soil microorganisms—a review. Soil Biology and Biochemistry, 42(12), 2058–2067.

[emi413220-bib-0008] Hutchins, D.A. & Capone, D.G. (2022) The marine nitrogen cycle: new developments and global change. Nature Reviews. Microbiology, 20(7), 401–414. 35132241 10.1038/s41579-022-00687-z

[emi413220-bib-0009] Hyatt, D. , Chen, G.‐L. , Locascio, P.F. , Land, M.L. , Larimer, F.W. & Hauser, L.J. (2010) Prodigal: prokaryotic gene recognition and translation initiation site identification. BMC Bioinformatics, 11, 119. 20211023 10.1186/1471-2105-11-119 PMC2848648

[emi413220-bib-0010] Javelle, A. , Lupo, D. , Zheng, L. , Li, X.‐D. , Winkler, F.K. & Merrick, M. (2006) An unusual twin‐His arrangement in the pore of ammonia channels is essential for substrate conductance. The Journal of Biological Chemistry, 281(51), 39492–39498. 17040913 10.1074/jbc.M608325200

[emi413220-bib-0011] Jiménez‐Gómez, C.P. & Cecilia, J.A. (2020) Chitosan: a natural biopolymer with a wide and varied range of applications. Molecules, 25(17), 3981. 32882899 10.3390/molecules25173981 PMC7504732

[emi413220-bib-0012] Jørgensen, N.O.G. , Kroer, N. , Coffin, R.B. & Hoch, M.P. (1999) Relations between bacterial nitrogen metabolism and growth efficiency in an estuarine and an open‐water ecosystem. Aquatic Microbial Ecology, 18, 247–261.

[emi413220-bib-0013] Katoh, K. , Misawa, K. , Kuma, K.‐I. & Miyata, T. (2002) MAFFT: a novel method for rapid multiple sequence alignment based on fast Fourier transform. Nucleic Acids Research, 30(14), 3059–3066. 12136088 10.1093/nar/gkf436 PMC135756

[emi413220-bib-0014] Kirchman, D.L. , Keil, R.G. & Wheeler, P.A. (1989) The effect of amino acids on ammonium utilization and regeneration by heterotrophic bacteria in the subarctic Pacific. Deep Sea Res A, 36(11), 1763–1776.

[emi413220-bib-0015] Kleiner, D. (1981) The transport of NH_3_ and NH_4_ ^+^ across biological membranes. Biochimica et Biophysica Acta, 639(1), 41–52. 7030397 10.1016/0304-4173(81)90004-5

[emi413220-bib-0016] Landgraf, A.J. & Lee, Y. (2015) Dimensionality reduction for binary data through the projection of natural parameters. Journal of Multivariate Analysis, 180, 104668.

[emi413220-bib-0017] Mooshammer, M. , Wanek, W. , Jones, S.H. , Richter, A. & Wagner, M. (2021) Cyanate is a low abundance but actively cycled nitrogen compound in soil. Communications Earth & Environment, 2(1), 1–10.

[emi413220-bib-0018] Mulholland, M.R. , Ohki, K. & Capone, D.G. (1999) Nitrogen utilization and metabolism relative to patterns of N_2_ fixation in cultures of *Trichodesmium* nibb1067. Journal of Phycology, 35(5), 977–988.

[emi413220-bib-0019] Nayfach, S. , Roux, S. , Seshadri, R. , Udwary, D. , Varghese, N. , Schulz, F. et al. (2021) A genomic catalog of Earth's microbiomes. Nature Biotechnology, 39(4), 499–509. 10.1038/s41587-020-0718-6 PMC8041624 33169036

[emi413220-bib-0020] Noh, S. (2021) Linear paths to genome reduction in a defensive symbiont. Proc Natl Acad Sci U S A, 118(22), e2106280118. 33975871 10.1073/pnas.2106280118 PMC8179129

[emi413220-bib-0021] Norman, J.S. & Friesen, M.L. (2017) Complex N acquisition by soil diazotrophs: how the ability to release exoenzymes affects N fixation by terrestrial free‐living diazotrophs. The ISME Journal, 11(2), 315–326. 27898052 10.1038/ismej.2016.127 PMC5270568

[emi413220-bib-0022] Price, M.N. , Dehal, P.S. & Arkin, A.P. (2009) FastTree: computing large minimum evolution trees with profiles instead of a distance matrix. Molecular Biology and Evolution, 26(7), 1641–1650. 19377059 10.1093/molbev/msp077 PMC2693737

[emi413220-bib-0023] Price, M.N. , Dehal, P.S. & Arkin, A.P. (2010) FastTree 2—approximately maximum‐likelihood trees for large alignments. PLoS One, 5(3), e9490. 20224823 10.1371/journal.pone.0009490 PMC2835736

[emi413220-bib-0024] Rajeev, L. , Chen, A. , Kazakov, A.E. , Luning, E.G. , Zane, G.M. , Novichkov, P.S. et al. (2015) Regulation of nitrite stress response in D*esulfovibrio vulgaris* hildenborough, a model sulfate‐reducing bacterium. Journal of Bacteriology, 197(21), 3400–3408. 26283774 10.1128/JB.00319-15 PMC4621071

[emi413220-bib-0025] Saier, M.H., Jr. , Tran, C.V. & Barabote, R.D. (2006) TCDB: the transporter classification database for membrane transport protein analyses and information. Nucleic Acids Research, 34, D181–D186. 16381841 10.1093/nar/gkj001 PMC1334385

[emi413220-bib-0026] Saier, M.H., Jr. (2000) Families of transmembrane transporters selective for amino acids and their derivatives. Microbiology, 146, 1775–1795. 10931885 10.1099/00221287-146-8-1775

[emi413220-bib-0027] Saier, M.H. , Reddy, V.S. , Moreno‐Hagelsieb, G. , Hendargo, K.J. , Zhang, Y. , Iddamsetty, V. et al. (2021) The transporter classification database (TCDB): 2021 update. Nucleic Acids Research, 49(D1), D461–D467. 33170213 10.1093/nar/gkaa1004 PMC7778945

[emi413220-bib-0028] Sanderson, T. (2022) Taxonium, a web‐based tool for exploring large phylogenetic trees. Elife, 11, e82392. 36377483 10.7554/eLife.82392 PMC9704803

[emi413220-bib-0029] Smith, E.M. , Vargas, R. , Guevara, M. , Tarin, T. & Pouyat, R.V. (2022) Spatial variability and uncertainty of soil nitrogen across the conterminous United States at different depths. Ecosphere, 13(7), e4170.

[emi413220-bib-0030] Tripp, H.J. , Bench, S.R. , Turk, K.A. , Foster, R.A. , Desany, B.A. , Niazi, F. et al. (2010) Metabolic streamlining in an open‐ocean nitrogen‐fixing cyanobacterium. Nature, 464(7285), 90–94. 20173737 10.1038/nature08786

[emi413220-bib-0031] UniProt Consortium . (2023) UniProt: the universal protein knowledgebase in 2023. Nucleic Acids Research, 51(D1), D523–D531. 36408920 10.1093/nar/gkac1052 PMC9825514

