# Supplementary material for: A genomic view of environmental and life history controls on microbial nitrogen acquisition strategies
Source: Environ Microbiol Rep. 2023 Dec 6;16(1):e13220. doi: 10.1111/1758-2229.13220 (PMC10866080; doi:10.1111/1758-2229.13220)
Supplement: Supplementary file 1 — DATA S1: Supporting Information. [file EMI4-16-e13220-s001.pdf]

A Genomic View of Environmental and Life History Controls on Microbial Nitrogen Acquisition Strategies

Linta Reji*[1,2], Romain Darnajoux[1], Xinning Zhang*[1,2]
[1,2]Department of Geosciences, Princeton University, Princeton NJ
[1,2] High Meadows Environmental Institute, Princeton University, Princeton NJ

*corresponding authors

Supplementary Figures

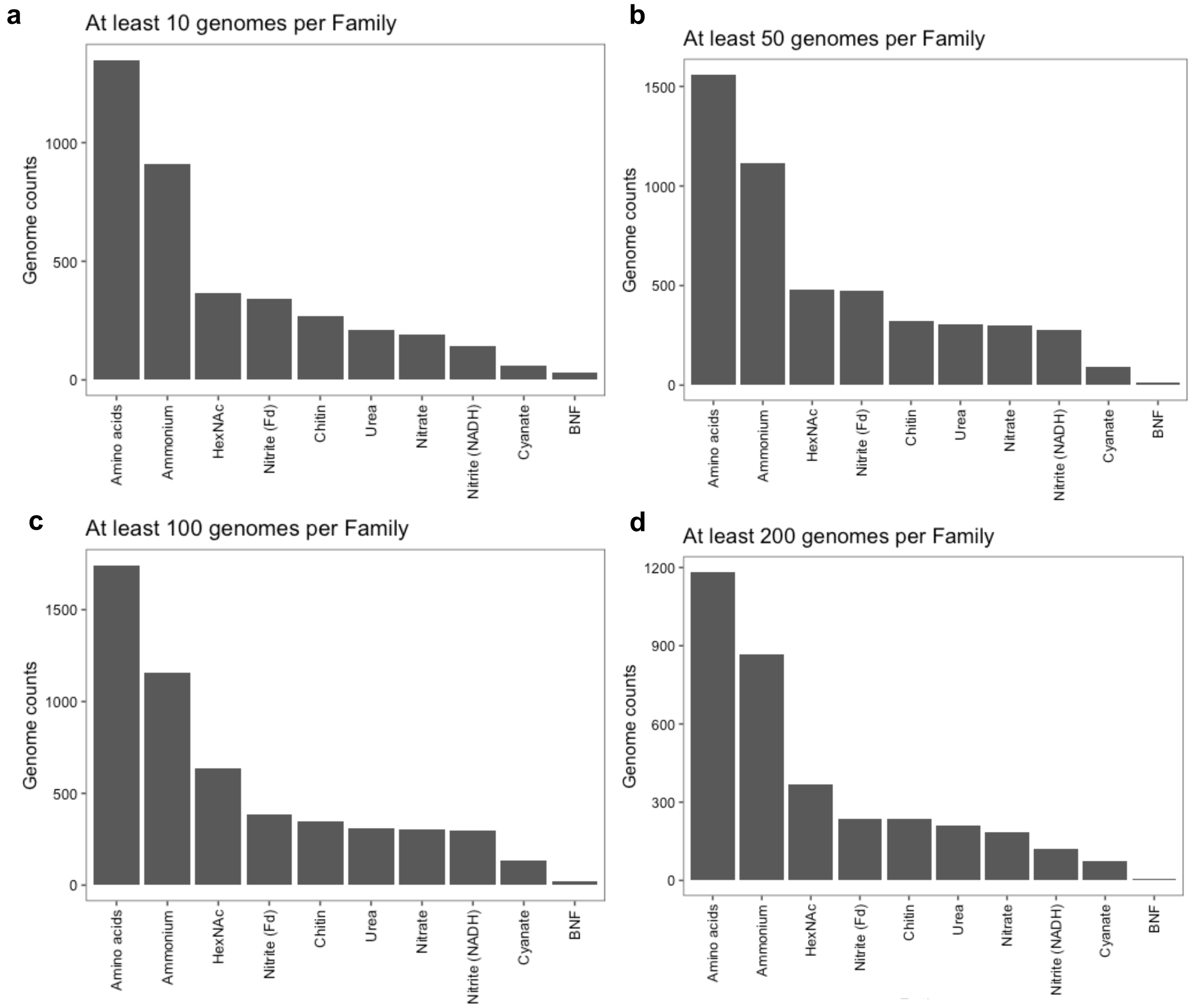

Figure S1: Prevalence of the various N-compound acquisition strategies among genomes in the filtered GEMs dataset, with different sample sizes. The dataset was filtered to retain family-level lineages that contain at least 10, 50, 100, or 200 genomes per family.

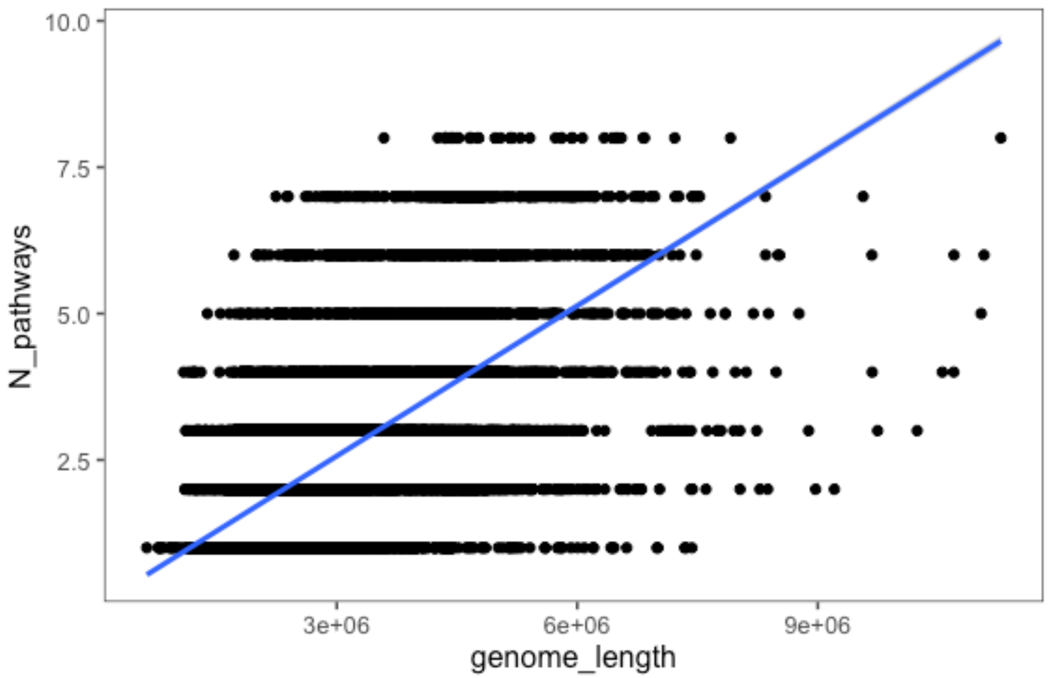

Figure S2: Correlation between genome length and the number of total N assimilation strategies.

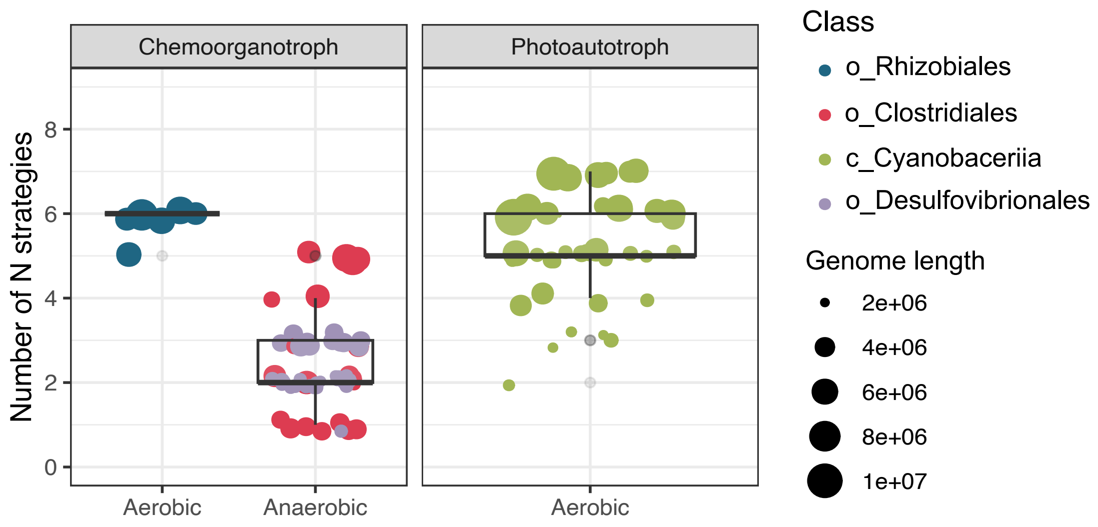

Figure S3. Total number of N-acquisition pathways in the GEMs dataset corresponding to the selected clades presented in Figure 3a, partitioned by metabolic mode. The filtered GEMs set did not include any methanogens.

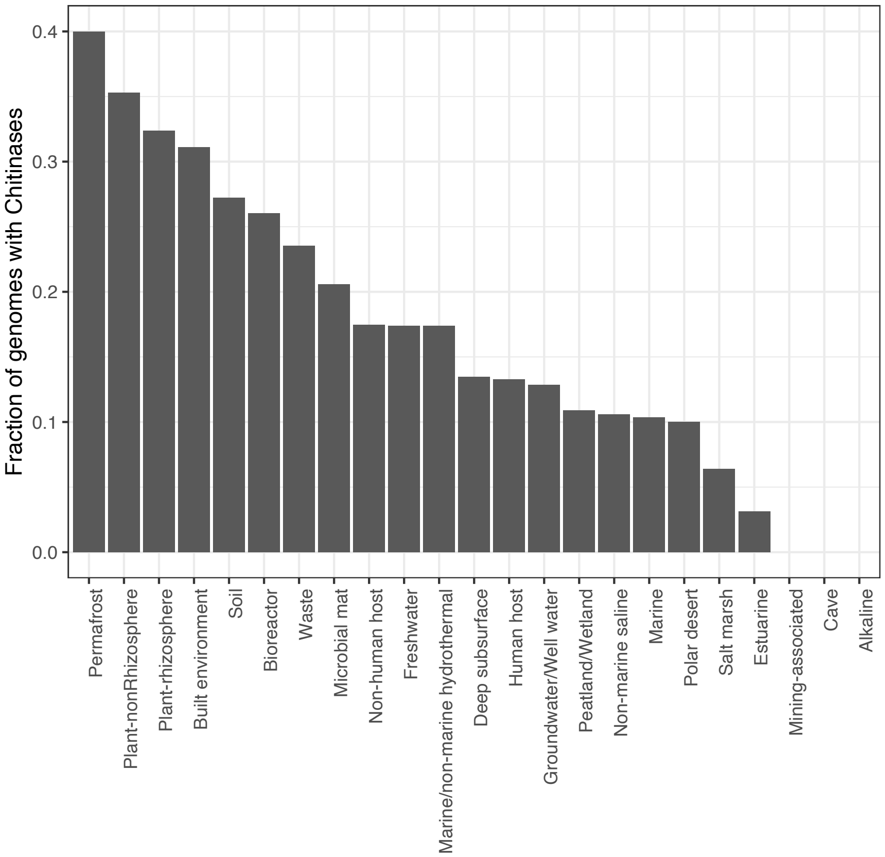

Figure S4: Relative counts of genomes with chitinases across habitat types.
